# The Effect of acetyl-L-carnitine, Alpha-lipoic Acid, and Coenzyme Q10 Combination in Preventing Anti-tuberculosis Drug-induced Hepatotoxicity: A Randomized, Double-blind, Placebo-controlled Clinical Trial

**DOI:** 10.22037/ijpr.2021.114618.14953

**Published:** 2021

**Authors:** Reza Hakimizad, Rasool Soltani, Farzin Khorvash, Majid Marjani, Farzaneh Dastan

**Affiliations:** a *Students Research Committee, School of Pharmacy and Pharmaceutical Sciences, Isfahan University of Medical Sciences, Isfahan, Iran. *; b *Department of Clinical Pharmacy and Pharmacy Practice, School of Pharmacy and Pharmaceutical Sciences, Isfahan University of Medical Sciences, Isfahan, Iran. *; c *Infectious Diseases and Tropical Medicine Research Center, Isfahan University of Medical Sciences, Isfahan, Iran. *; d *Nosocomial Infection Research Center, Isfahan University of Medical Sciences, Isfahan, Iran. *; e *Clinical Tuberculosis and Epidemiology Research Center, National Research Institute of Tuberculosis and Lung Diseases (NRITLD), Shahid Beheshti University of Medical Sciences, Tehran, Iran. *; f *Chronic Respiratory Disease Research Center, National Research Institute of Tuberculosis and Lung Disease (NRITLD), Shahid Beheshti University of Medical Sciences, Tehran, Iran. *; g *Department of Clinical Pharmacy, School of Pharmacy, Shahid Beheshti University of Medical Sciences, Tehran, Iran.*

**Keywords:** Anti-tuberculosis drugs, Hepatotoxicity, Acetyl-L-carnitine, Alpha-lipoic acid, Coenzyme Q10

## Abstract

Drug-induced liver injury (DILI) is one of the most serious adverse effects of anti-tuberculosis (TB) drugs. A suggested mechanism of this adverse effect is mitochondrial dysfunction (MDF). The purpose of this study was an evaluation of the possible preventive effects of the combination of acetyl-L-carnitine (ALCAR), alpha-lipoic acid (ALA), and coenzyme Q10 (CoQ10), as mitochondrial nutrients (MNs), against anti-TB DILI. In this clinical trial, patients who met the inclusion criteria were randomly assigned to either experimental (n = 44) or placebo (n = 43) groups. The experimental group received capsules containing CoQ10 (200 mg) + ALA (250 mg) + ALCAR (250 mg) orally twice daily for two weeks, and the placebo group received oral placebo capsules with the same interval and duration. The mean serum levels of aspartate aminotransferase (AST), alanine aminotransferase (ALT), and total bilirubin (TBil) at the end of the first and second weeks as well as the incidence of DILI during the intervention were recorded and compared between the two groups. At the end of the study, the mean serum levels of AST and ALT in the experimental group were significantly lower than the placebo group (36.27 ± 36.43 *vs.* 86.02 ± 97.23 and 28.41 ± 27.41 *vs.* 78.80 ± 118.28, respectively, *P* = 0.003 for both). Also, the incidence of anti-TB DILI was significantly lower in the experimental group than the placebo group (6.8% *vs*. 25.6%, *P* = 0.017). In conclusion, using the combination of ALCAR, ALA, and CoQ10 may provide an effective strategy in preventing anti-TB DILI.

## Introduction

Tuberculosis (TB) is an important health problem in the world that is one of the top 10 causes of death. According to the World Health Organization (WHO) global TB report, the incidence of TB in Iran was about 11000 cases in 2017 (1).

Standard treatment of TB includes the combination of isoniazid (INH), rifampin (RIF), pyrazinamide (PZA), and ethambutol (EMB). The first three drugs are hepatotoxic, and drug-induced liver injury (DILI) is one of the most serious adverse effects among TB patients who receive this regimen (2). The reported incidence of anti-TB DILI has been from 5% to as high as 33%, depending on the DILI definition and the studied population (3). Anti-TB DILI may cause considerable morbidity or mortality as well as the discontinuation of the suspected agent(s), significantly contributing to non-adherence, treatment failure, relapse, and drug resistance (2). Therefore, any preventive measure for decreasing the risk of anti-TB DILI is crucial.

The exact mechanism of anti-TB DILI has not yet been completely understood; however, recent research suggests that mitochondrial dysfunction (MDF) may contribute to DILI. The elevated levels of RIF and INH reactive intermediates cause MDF and induce cellular oxidative stress (OS) due to reactive oxygen species (ROS) production. Thus, treatments that target OS/MDF may represent a novel therapeutic approach to the prevention of anti-TB DILI (4). 

Acetyl-L-Carnitine (ALCAR) is an effective substrate for mitochondrial energy metabolism and among carnitine derivatives, quantitatively and functionally is the most significant ester. Carnitine also has free radical scavenging activity improving antioxidant status (5, 6). The hepatoprotective effects of carnitine against several agents including acetaminophen, sodium valproate, and arsenic have been demonstrated in some studies (6-8). 

Alpha-Lipoic acid (ALA), also known as thioctic acid, functions as a coenzyme for mitochondrial enzymes and an amphipathic antioxidant (9, 10). Several experimental studies have suggested that ALA has beneficial effects against liver injury induced by acetaminophen, chloroquine, methotrexate, and alcohol (11). Moreover, the hepatoprotective effect of ALA against RIF+INH-induced hepatotoxicity in an animal model has been documented (12).

CoQ10 is an electron carrier in the mitochondrial respiratory chain. In addition to its key role in mitochondria, CoQ10 acts as the only endogenous lipid-soluble antioxidant with potent antioxidant properties, scavenging free radicals and inhibiting lipid peroxidation (13). Several studies in animal models have demonstrated the ability of CoQ10 to reduce or prevent acetaminophen hepatotoxicity as well as metabolic-stress-induced liver damage (14, 15). Furthermore, an animal study showed the protective effect of CoQ10 against RIF+INH-induced hepatotoxicity (16). 

Evidence from human trials or *in-vitro* studies show synergistic effects of ALCAR, ALA, and CoQ10 (two or three of these agents) for reduction of insulin resistance (17) and myocardial damage (18), rescuing disuse-induced muscle atrophy (19), and improving liver mitochondrial content, size, and serum liver enzymes in non-alcoholic fatty liver disease (20). The present study aimed to investigate the possible preventive effects of the combination of these agents against anti-TB DILI.

## Experimental

*Setting and study population*

This randomized, double-blind, multicenter, placebo-controlled clinical trial was conducted in two hospitals in Iran including Masih Daneshvari hospital, the national referral center of tuberculosis and lung diseases affiliated to Shahid Beheshti University of Medical Sciences, and Al-Zahra hospital complex affiliated to Isfahan University of Medical Sciences (IUMS), the largest tertiary care medical center and a referral infectious diseases ward in the region, from June 2016 to September 2018. The study protocol and procedures were in accordance with the ethical standards outlined in the Declaration of Helsinki and its later amendments (21) and approved by the ethical committee of IUMS (ethical code: IR.MUI.REC.1395.3.929). All participants were provided written informed consent before entering the study. The study was registered in the Iranian Registry of Clinical Trials with the registration number IRCT20150721023282N11. 

Participants were selected from inpatients in the above-mentioned hospitals and were included if they 1) aged over 18 years regardless of gender; 2) were diagnosed with pulmonary TB based on clinical, radiological (X-ray), and microbiological (at least two out of three consecutive sputum samples were positive for Acid Fast Bacilli) findings; 3) had no previous history of using anti-TB drugs; 4) had normal liver function tests (LFT) at baseline (alanine aminotransferase [ALT] < 35 U/L, aspartate aminotransferase [AST] < 35 U/L, alkaline phosphatase [ALP] < 130 U/L, and total bilirubin [TBil] < 1 mg/dL) (22).

Patients were excluded if they 1) had comorbid liver diseases (*e.g*., hepatitis B, hepatitis C, autoimmune hepatitis, and hepatic cirrhosis); 2) had a severe heart (e.g., heart failure and angina pectoris), kidney (serum creatinine more than 1.2 mg/dL), or gastrointestinal (inflammatory bowel disease and malabsorption) disorder; 3) were positive for HIV infection; 4) had the history of taking either ALCAR, ALA, or CoQ10 during the last 4 weeks; 5) had concomitant use of known hepatotoxic drugs (*e.g*., sodium valproate and methotrexate); 6) were allergic or intolerant to the agents; and 7) were pregnant or lactating.

*Study design and interventions*

The subjects were randomly assigned to either experimental or placebo groups (1:1 ratio). Both groups received standard anti-TB drug regimen including isoniazid (5 mg/kg), rifampin (10 mg/kg), ethambutol (15 mg/kg), and pyrazinamide (25 mg/kg) according to WHO standard regimen (1). Additionally, the experimental group received drug capsules containing CoQ10 (200 mg) + ALA (250 mg) + ALCAR (250 mg) (Vitacost^®^, USA) orally twice per day for two weeks, and the placebo group received oral placebo capsules with the same shape, color, size, and weight (manufactured by the pharmaceutical laboratory, School of Pharmacy and Pharmaceutical Sciences, IUMS) twice daily for two weeks. The drug and placebo capsules were started concomitantly with the anti-TB regimen. For randomization and blindness, each capsule container was given a unique code based on its content (drug or placebo) by a third person unaware of the study design. Upon inclusion of each patient, a container was randomly given to him/her, with the code being recorded on his/her consent form. At the end of the study and after the determination of the patient’s own results, the recorded code was identified in terms of intervention type. Therefore, the prescribing physician, the patients, the data collector, and laboratory personnel were all blinded to the type of intervention.

*Assessments and outcome measures*

LFTs were determined at the start of treatment and weekly for two weeks by measuring serum aspartate aminotransferase (AST), alanine aminotransferase (ALT), and total bilirubin (TBil). Serum albumin was measured only at baseline for evaluation of hypoalbuminemia as a possible risk factor for DILI. Patients were monitored every day for any symptom or sign related to adverse drug effects.

Anti-TB DILI was diagnosed by the presence of at least one of the following criteria based on the definition of the American Thoracic Society (ATS): 1) an increase in serum ALT greater than three times the upper limit of normal (ULN), concomitant with symptoms of hepatic toxicity consisting nausea, vomiting, abdominal pain, weakness, and jaundice; 2) a rise of more than five times the ULN in serum ALT with or without liver injury symptoms (23). 

All patients with DILI were divided into three following subgroups according to WHO Toxicity Classification Standards (23): Severe (grade 4) DILI, the elevation of peak serum ALT > 10 times the ULN; moderate (grade 3) DILI, the elevation of peak ALT > 5 to ≤ 10 times the ULN; mild DILI (grade 2), the elevation of peak ALT > 2.5 to ≤ 5 times the ULN. The ULN of ALT in our study was 40 U/L. Of note, grade 1 (ALT < 2.5 times the ULN) was not included in this study due to its less importance. Once DILI was detected, the change of anti-TB treatment was considered according to the ATS guideline, including drug replacement, interruption, discontinuation, and dose reduction (23). 

The primary outcome measures were the differences between the two groups regarding the mean values of LFTs at the end of the first and second weeks, as well as the number of cases with DILI during the intervention. The secondary outcome measure was the frequency of any adverse effect reported by the patients during the study. 

*Statistical analysis*

Data were analyzed using SPSS version 22.0 (SPSS Inc., Chicago, IL, USA) for Windows. The distribution pattern of continuous data was evaluated by the Kolmogorov–Smirnov test. Independent samples t-test and Mann–Whitney U-test were used for comparing means ± SD of normally (ALP levels in all time points) and non-normally (all other continuous variables) distributed continuous variables, respectively. Chi-square or Fisher’s exact tests were used to analyze probable associations between categorical variables. To find the probable association of evaluated variables with anti-TB DILI, logistic regression analysis was performed with the results being represented as odds ratio (OR) and their 95% confidence intervals (CIs). For this, the occurrence of anti-TB DILI was considered as a dependent variable, while the patients’ demographic, clinical, and laboratory data were assumed as independent variables. First, the association of each independent variable was evaluated separately using univariate analysis followed by multivariate analysis of variables significantly associated with anti-TB DILI. A *P-*value less than 0.05 was considered statistically significant.

## Results

*Participants*

**Figure 1:**
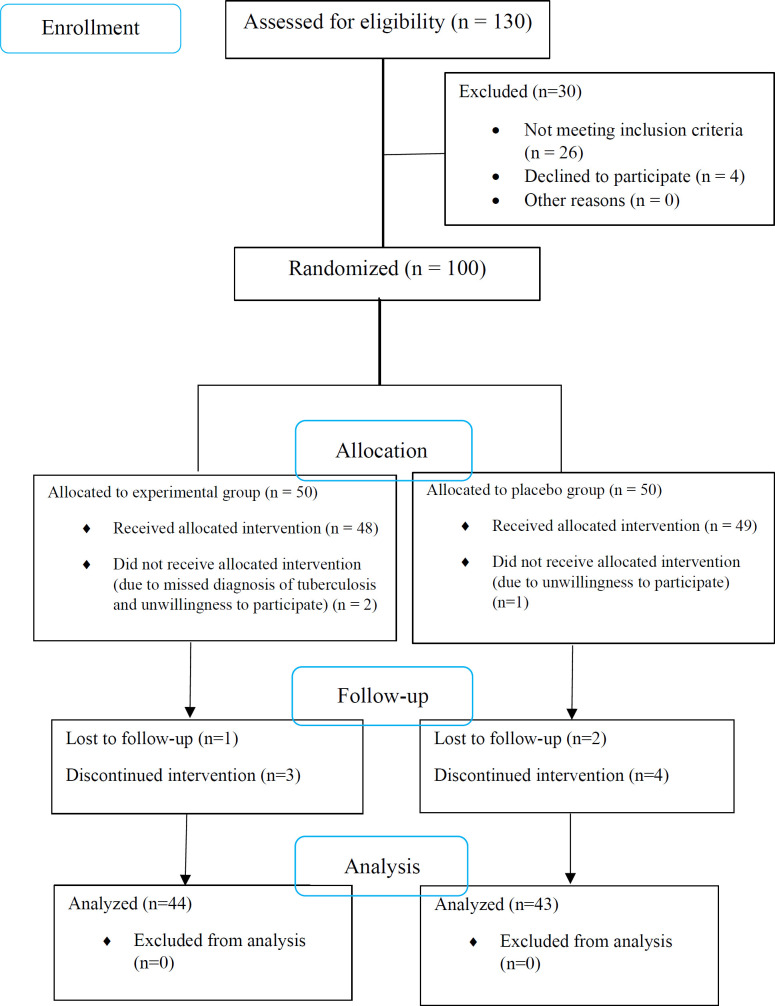
CONSORT flowchart of the study

From 130 patients assessed for eligibility, a total of 100 confirmed cases of TB underwent randomization, of whom 3 patients were excluded due to unwillingness to participate. Of 97 patients who underwent the interventions, 4 and 6 patients were excluded from the experimental and placebo groups, respectively, because of either no follow-up or discontinuation of the use of capsules (Figure 1). Finally, 44 patients in the experimental group and 43 patients in the placebo group completed the study. All of the cases had pulmonary TB. There was no significant difference between the two groups regarding demographic and clinical characteristics (Table 1). 

Baseline levels of ALT, AST, TBil, and ALP were not statistically different between the groups (Table 1). Although the mean values of ALP and TBil after 1 and 2 weeks of treatment were not significantly different between the two groups, mean levels of AST and ALT were significantly higher in the placebo group than in the experimental group after 2 weeks of treatment (*P *= 0.003).

*Incidence of liver injury*

The incidence of DILI in the experimental group (n = 3, 6.8%) was significantly lower than that in the placebo group (n = 11, 25.6%; *P* = 0.017). As shown in Table 2, most cases of DILI in the placebo group were mild.

*Associated factors of anti-TB DILI*

According to univariate analysis, hypoalbuminemia (serum albumin less than 3.5 g/dL) and being in the experimental group were significantly related to anti-TB DILI (Table 3). After adjusting these variables in the multivariate logistic regression model, hypoalbuminemia (odds ratio [OR] 10.07, 95% confidence interval [CI] 1.23–82.66, *P* = 0.032) and being in the experimental group (OR = 0.25, 95% CI = 0.06–0.99, *P* = 0.049) remained statistically significant, showing that the rate of anti-TB DILI was significantly higher in patients with hypoalbuminemia and lower in the experimental group (patients who received CoQ10/ALA/ALCAR capsules) than the placebo group. 

*Adverse drug reactions*

During the study period, no significant adverse reactions relevant to the experimental group were detected. However, mild nausea, abdominal cramps, and dyspepsia were reported in two (4.5%) patients of this group. Furthermore, no patients discontinued the drugs because of these adverse effects. 

*Effect size and study power*

Using ALT serum levels at the end of the intervention, the effect size (d) and study power were calculated as 0.43 and 77.7%, respectively. 

## Discussion

Anti-TB DILI is the primary cause of treatment discontinuation, change of the regimen, reduced efficacy, and drug resistance (3, 24). The overall incidence rate of DILI among our study population receiving the WHO-recommended TB regimen for pulmonary TB was 16.1% which was similar to the reported rate of previous studies in Iran (25, 26). This clinical trial found a significant difference in DILI incidence between MNs treatment consisting of ALCAR, ALA, and CoQ10, and placebo (*P* = 0.017). Furthermore, in the subgroups of the severity of the liver injury, we found a significant difference between the two groups in terms of mild DILI (*P* = 0.03), which was the most frequent type of liver injury in ours as previous studies (24, 27). This result demonstrates that our combination was more effective in the prevention of mild to moderate DILI rather than moderate to severe liver injury. According to the logistic regression analysis, the risk of DILI incidence in the experimental group was 0.25 times that of the placebo group indicating that the prophylactic use of this compound could prevent DILI.

 ALT and AST are reliable markers of liver function and are known to be elevated in a variety of clinical conditions associated with liver damage (28). Regarding the results of LFTs, patients of the experimental group had significantly lower levels of ALT (~28 *vs*. 79 IU/L, *P* = 0.003) and AST (~36 *vs.* 86 IU/L, *P* = 0.003) compared to those of the placebo group at week 2 of treatment. This shows the potential of our experimental compound for inhibition of the elevation of liver enzymes resulting from liver injury.

Many risk factors for DILI have been reported. Among the most widely accepted risk factors are advanced age (above 60 years), female sex, slow acetylator status, malnutrition, HIV co-infection, and pre-existent liver disease. In the present study, hypoalbuminemia (serum albumin less than 3.5 g/dL) was detected as a risk factor for anti-TB-induced hepatotoxicity, which is supported by some other reports (29, 30).

 Although anti-TB drugs have been used for decades worldwide, the mechanism of ant-TB DILI is not fully understood. It has been suggested that OS/MDF may contribute to anti-TB DILI. Mitochondria are important targets in DILI. Free radical generation, loss of membrane potential, permeability transition, altered dynamics, and impaired biogenesis are the mitochondrial effects of RIF+INH. Though, it seems that the common process in drug-induced mitochondrial dysfunction is increased production of ROS, leading to induction of the mitochondrial permeability transition and triggering either an immune response or hepatic apoptosis/necrosis and subsequent cell death. Besides, recent evidence shows that mitochondrial perturbations also influence the recovery of hepatic function. In conclusion, DILI is associated with OS/MDF. Thus, mitochondrial-targeted treatments termed MN, including carnitine (or its derivatives), ALA, and CoQ10, may represent a novel therapeutic approach to prevent liver injury (4, 31). 

Carnitine performs several essential functions, including transport of fatty acids, stabilization of cell membranes, and regulation of the mitochondrial acyl-CoA/CoA ratio. Also, it has been shown to be a specific inhibitor of mitochondrial reactive oxygen species. Carnitine derivatives reduce C-reactive protein (CRP) and tumor necrosis factor-alpha (TNF-α), consequently improving liver function. ALCAR is more often used in research than L-carnitine, because it has more absorption through the small intestine and more passage across the blood-brain barrier (32, 33). Hamza *et al.* and Malaguarnera *et al.* have reported that the administration of carnitine could improve nonalcoholic fatty liver disease and nonalcoholic steatohepatitis. They suggested that carnitine could decrease the liver enzymes by reducing beta-oxidation and limiting oxidative stress in the mitochondria as well as modulating inflammatory response (33, 34). Lheureux *et al*. suggested that carnitine helps to protect the cell from the membrane-destabilizing effects of toxic acyl groups and, therefore, prevents intramitochondrial accumulation (7). Moreover, the potential hepatoprotective effect of carnitine against TB drugs has been demonstrated in one clinical study performed by Hatamkhani *et al. *in which the authors suggested that carnitine could improve liver function by decreasing oxidative stress, increasing free radical scavenging, improving mitochondrial function, and modulation of lipid peroxidation (35).

ALA functions both as a coenzyme for mitochondrial enzymes and as an ideal antioxidant. It can scavenge a number of free radicals, including ROS, regenerate endogenous antioxidants such as CoQ10 and glutathione, reduce lipid oxidation, and repair oxidatively damaged biomolecules. Therefore, it can be used to prevent or treat several pathological conditions mediated via oxidative stress and mitochondrial dysfunction (14, 36). Pari and Murugavel reported that, compared to silymarin as a hepatoprotective reference agent, orally administered ALA against chloroquine-induced hepatotoxicity in rats showed significantly improved levels of plasma antioxidants and GSH as well as decreased serum levels of AST, ALT, ALP, and TBil (37). 

Considering the matched groups of our study, we can suggest that 2 weeks of adjuvant mitochondrial nutrients supplementation consisting of ALCAR, ALA, and CoQ10 has contributed to less development of anti-TB DILI. This combination may have a potential hepatoprotective property during the short-term treatment of anti-TB drugs.

The main limitations of our study were the short duration of the intervention, relatively small sample size, and lack of assessment of several other liver function tests, including serum albumin and international normalized ratio (INR). 

In conclusion, based on the results, we propose that using optimal combinations of MNs including ALCAR, ALA, and CoQ10 to target OS/MDF may provide an effective strategy in preventing anti-TB DILI. Therefore, the combination could be considered as a potential supplement for patients receiving anti-TB agents. However, additional studies with larger sample sizes and longer duration are necessary to evaluate the exact effect of these MNs among special high-risk groups in the prevention of DILI.

**Table 1:** Baseline demographic, clinical, and laboratory characteristics of study patients

**Characteristics**	**Placebo group** **(n** **= 43)**	**Experimental group** **(n = 44)**	***P-*** **value**
Age (years)	54.5 ± 16.5	52.9 ± 16.1	0.66
Sex			0.60
Female	21 (48.8)	19 (43.2)	
Male	22 (51.2)	25 (56.8)	
Nationality			0.51
Iranian	36 (83.7)	39 (88.6)	
Non-Iranian	7 (16.3)	5 (11.4)	
Rural residence	15 (34.9)	16 (36.4)	0.89
Diabetes mellitus	7 (16.3)	5 (11.4)	0.51
Hypoalbuminemia	29 (67.4)	23 (52.3)	0.15
Smoking	8 (18.6)	6 (13.6)	0.53
**Baseline**			
ALT (IU/L)	21.91 ± 9.53	21.59 ± 10.09	0.88
AST (IU/L)	26.47 ± 10.43	26.16 ± 8.83	0.99
ALP (IU/L)	118.86 ± 50.49	111.27 ± 25.27	0.71
TBil (mg/dL)	0.80 ± 0.26	0.76 ± 0.27	0.59
**Week 1**			
ALT (IU/L)	61.77 ± 130.34	26.14 ± 20.30	0.06
AST (IU/L)	58.60 ± 87.72	33.43 ± 29.03	0.10
ALP (IU/L)	175.28 ± 52.36	148.18 ± 35.64	0.54
TBil (mg/dL)	0.86 ± 0.58	0.8 ± 0.47	0.73
**Week 2**			
ALT (IU/L)	78.80 ± 118.28	28.41 ± 27.31	0.003
AST (IU/L)	86.02 ± 97.23	36.27 ± 36.43	0.003
ALP (IU/L)	213.67 ± 53.84	194.75 ± 41.54	0.44
TBil (mg/dL)	1.08 ± 0.59	0.98 ± 0.51	0.52

**Table 2:** Comparison of the occurrence of liver injury in the two groups. All variables are expressed as frequency (%).

**Grading**	**Total patients** **(n** **= 87)**	**Placebo group** **(n** **= 43)**	**Experimental group** **(n** **= 44)**	***P-*** **value**
Normal liver function	53 (60.9)	20 (46.5)	33 (75)	0.006
Abnormal liver function	20 (23)	12 (27.9)	8 (18.2)	0.281
DILI	14 (16.1)	11 (25.6)	3 (6.8)	0.017
Mild liver injury	8 (9.2)	7 (16.3)	1 (2.3)	0.030
Moderate liver injury	4 (4.6)	3 (7)	1 (2.3)	0.360
Severe liver injury	2 (2.3)	1 (2.3)	1 (2.3)	1

DILI: drug-induced liver injury.

**Table 3:** The association of baseline characteristics of patients with DILI according to univariate logistic regression analysis

**Characteristics**	**Without DILI ** **(n = 73)**		**With DILI ** **(n = 14)**	***P-*** **value**		**OR**	**95% CI for OR**	
							**Lower**	**Upper**
Age (years)	53.67 ± 16.3	53.71 ± 16.6		0.99	1.00		0.97	1.03
Female	32 (43.8)	8 (57.1)		0.36	1.71		0.54	5.42
Iranian Nationality	64 (87.7)	11 (78.6)		0.37	1.94		0.45	8.31
Rural residence	29 (39.7)	2 (14.3)		0.09	0.25		0.05	1.21
Diabetes mellitus	8 (11)	4 (28.6)		0.09	3.25		0.82	12.82
Hypoalbuminemia	39 (53.4)	13 (92.9)		0.02	11.33		1.41	91.20
Smoking	12 (16.4)	2 (14.3)		0.84	0.85		0.17	4.28
Experimental group	41 (56.2)	3 (21.4)		0.026	0.21		0.06	0.83
Placebo group	32 (43.8)	11 (78.6)		0.026	4.70		1.21	18.26

Age is expressed as mean ± SD; the rest or variables are expressed as frequency (%). DILI: drug-induced liver injury; CI: confidence interval;

OR: odds ratio; SD: standard deviation.

## Author contributions

RS gave the study idea and designed it, and corrected the drafted manuscript. RH collected the data, performed the statistical analysis, and drafted the manuscript. FK and MM selected the eligible patients according to the inclusion criteria and followed them regarding the outcome measures. FD followed the patients in Masih Daneshvari hospital and corrected the drafted manuscript. 
